# The effect of impaction and a bioceramic coating on bone ingrowth in porous titanium particles: A bone chamber study in goats

**DOI:** 10.3109/17453674.2011.579515

**Published:** 2011-07-08

**Authors:** Lucas H Walschot, Barend W Schreurs, Nico Verdonschot, Pieter Buma

**Affiliations:** ^1^Orthopaedic Research Laboratory, Department of Orthopaedics, Radboud University Nijmegen Medical Centre, Nijmegen; ^2^Laboratory of Biomechanical Engineering, University of Twente, Enschede, the Netherlands

## Abstract

**Background and purpose:**

Porous titanium (Ti) particles can be impacted like cancellous allograft bone particles, and may therefore be used as bone substitute in impaction grafting. We evaluated the effect of impaction and of a thin silicated biphasic calcium phosphate coating on osteoconduction by Ti particles.

**Methods:**

The bone conduction chamber of Aspenberg was used in goats and filled with various groups of coated or uncoated small Ti particles (diameter 1.0–1.4 mm). Impacted allograft bone particles and empty chambers were used in control groups. Fluorochromes were administered at 4, 8, and 12 weeks. Maximum bone ingrowth distance was evaluated by histomorphometric analysis.

**Results:**

Histology of Ti particle graft cylinders showed a dense matrix with narrow inter-particle and intra-particle pores (< 100 μm), occluding the lumen of the bone chamber. Bone ingrowth distances gradually increased with time in all groups. Maximum bone ingrowth distance was higher in originally empty chambers than those with allograft bone particles (p = 0.01) and Ti particles (p < 0.001). Maximum bone ingrowth in allograft bone particles was higher than in all Ti groups (p **≤** 0.001). Impaction reduced osteoconduction and the coating partially compensated for the negative effect of impaction, but these differences were not statistically significant. No osteolytic reactions were found.

**Interpretation:**

Osteoconduction in the bone conduction chamber was reduced more by the insertion of small Ti particles than by insertion of small allograft bone particles. The osteoconductive potential of porous Ti particles should be studied further with larger-sized particles, which may allow bone ingrowth after impaction through larger inter-particle pores.

Allograft bone impaction grafting restores the original bone stock (Halliday et al. 2003). Morselized cancellous allograft bone chips remain the gold standard material, but they have their limitations such as limited availability, risk of pathogen transmission, and religious considerations (Conrad et al. 1995, Galea et al. 1998). Calcium phosphate particles have been used successfully with good long-term clinical results (Oonishi et al. 2008). However, bioceramics show inferior handling characteristics (Bolder et al. 2002, van Haaren et al. 2005), and accelerated resorption could compromise construct stability (Ninomiya et al. 2001).

Porous commercially pure titanium (Ti) particles are not resorbed by osteoclasts; thus, the stability of the graft layer is not reduced by remodeling. Long-term stability is probably dependent on ingrowth of fibrous tissue and bone. A canine model and a human retrieval model have shown that non-impacted, non-coated small Ti particles are osteoconductive when they are used in the femur in combination with an uncemented, vibration-based technique to insert the stem into the bed of Ti particles (Alffram et al. 2007, Turner et al. 2007). We intend to use larger and highly porous Ti particles with a different surgical technique: cemented impaction grafting of the acetabulum and femur. Impaction of large Ti particles creates a graft layer with good entanglement and primary stability (Aquarius et al. 2009, Walschot et al. 2010). However, we do not know whether impacted Ti particles allow tissue ingrowth like non-impacted Ti particles: impaction poses a threat to the osteoconduction of a non-degradable material like Ti particles by obliteration of ingrowth spaces, which is even observed with a resorbable material like allograft bone (Tägil and Aspenberg 1998, Jeppsson et al. 2003). Thus, we evaluated the effect of impaction on osteoconduction by small Ti particles in a bone conduction chamber (Aspenberg et al. 1996) in goats. We expected that the addition of a thin sol-gel-formed silicated calcium phosphate coating would favor osteoconduction of this porous titanium graft material (Nguyen et al. 2004).

## Animals and methods

### Animals

12 mature Dutch milk goats (Capra hircus sana) with a mean weight of 47 kg (range 38–59) were provided by the Central Animal Laboratory of the University of Nijmegen, the Netherlands. The animals were housed together in a climatologically controlled room at least 1 week before to surgery (tenderfoot bottom, 18–22°C, humidity 60%) and provided with fresh hay, concentrate, pulp, and water.

### Implanted materials

**Figure 1.**
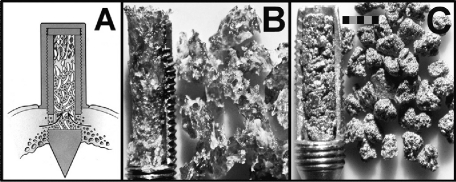
A. Cross-sectional drawing of an implanted bone chamber in the proximal tibia of the goat. B. Cylinder of impacted bone particles in an opened bone chamber (left) with the corresponding amount of graft before impaction (right). C. Cylinder of impacted titanium particles (left) with the corresponding amount of graft before impaction (right). Scale bar represents 2 mm.

The bone conduction chamber of Aspenberg is a hollow screw (lumen: 2 mm in diameter, 7 mm high) made of commercially pure titanium with 2 ingrowth openings at the tip (Aspenberg et al. 1996). It consists of 2 half-cylinders held together by a hexagonal cap with a 1-mm thick disk placed under the cap of the bone chamber to place the ingrowth openings at the level of the endostium of the goat tibia (Figure 1). 6 groups of bone conduction chambers were implanted (Table). A pool of mixed cancellous allograft bone particles was obtained by nibbling the freshly frozen (–40°C) sternums of 5 goats with a rongeur to chips of about 1 × 1 × 2 mm. Microbial culture was used to exclude contamination. After impaction, specimens were stored frozen (–40°C) under sterile conditions and thawed before implantation. Empty bone chambers were used in a control group.

**Table:** Groups implanted, corresponding graft materials, and volume fractions (mean, SD) from cross-sectional views of the graft cylinder

Group	Particulate material	Graft porosity (%)
Empty	No material inserted	Not applicable
Bone	Impacted allograft cancellous bone	0.39, 0.09
Ti	Titanium	0.78, 0.05
Ti_imp	Titanium, impacted	0.56, 0.02
Ti_coat	Titanium particles, with CaPO_4_ coating	0.79, 0.05
Ti_imp_coat	Titanium, impacted, with CaPO_4_ coating	0.60, 0.05

Porous titanium particles consist of commercially pure titanium (Hereford Metal Powder Company Ltd., Hereford, UK). The interconnected porosity of non-impacted individual Ti particles was 74% (SD 4). Small Ti particles with a diameter of 1.0–1.4 mm were used. (The particles passed a sieve with pores of 1.4 mm but were stopped by a sieve with pores of 1.0 mm). 4 different groups of Ti particles were implanted: “Ti”: not impacted, not coated; “Ti_imp”: impacted, not coated; “Ti_coat”: not impacted, coated; and “Ti_imp_coat”: impacted, coated. After sieving, the Ti particles were cleaned in a standardized manner to remove potential particulate and chemical pollution (procedure number PS03-016 CAM Implants BV, Leiden, the Netherlands). Sieved and cleaned Ti particles were either sterilized in an autoclave prior to (non-coated) in vivo use or processed further by application of a Bonitmatrix coating (DOT GmbH, Rostock, Germany). Bonitmatrix is a commercially available silicated biphasic calcium phosphate, which is normally available as a granular material. The Bonitmatrix substance was applied to the Ti particles as a thin coating through a sol-gel procedure at low temperature (Teller et al. 2007). The coating consists of biphasic calcium phosphate (87%; HA/TCP 60:40) and silicium dioxide (13%); it increased the weight of the Ti particles by 3.5%. The surfaces of coated Ti particles were covered with numerous ceramic granules with a mean diameter of about 5 μm. Coated Ti particles were gamma-sterilized before implantation.

### Impaction procedure

Specimens of all graft groups were prepared by manual insertion of the corresponding graft materials into the lumen (diamater 2.0 mm) of the bone chamber. Allograft bone particles, Ti and Tci, were manually impacted with a sliding thread (diameter 1.8 mm), with a sliding weight of 9.8 g ending against a stopper at the foot of the thread. The foot of the thread was inserted into the upper lumen of the bone chamber (on top of the grafts, acting as an impactor) containing the graft particles during impaction in the rest of the lumen of the bone chamber. In order to standardize the impaction procedure, the weight was dropped 30 times from a height of 30 cm. In this way, manual impaction was mimicked—as used during previous in vitro testing (Walschot et al. 2010) (Figure 1). 5 non-implanted specimens were used for estimation of graft volume over total volume fraction from central longitudinal slices. Mineralized bone matrix area was determined from 20-μm thin non-decalcified sections by light microscopy after Goldner staining, and cross-sectional area of titanium was determined by backscatter electron imaging. Volume fractions of bone and titanium were calculated with interactive computer-controlled image analysis by dividing the area of mineralized bone matrix and the titanium matrix area by the whole graft cylinder area (Table).

### Surgical technique

The implant position on the tibia was randomized for every bone chamber in every goat by picking folded notes from a box, with the corresponding graft group written on the (hidden) inside of the note. 12 goats were anesthetized with pentobarbital (1,200 mg) and isoflurane. A longitudinal incision over the tibia was used to expose the proximal anteromedial metaphysis. A measuring device and k-wires were used to fixate a drilling and tapping guide block, which enabled standardized positioning of 6 bone chambers in the tibia with a distance of at least 12 mm between chambers. After drilling and tapping of the screw holes, a round biopsy punch (6 mm in diameter) was used for excision of periosteum around the screw hole to enable direct contact between the cap of the bone chamber and the tibial cortex. Each goat received 6 bone chambers in the right tibia and 6 bone chambers in the left tibia. As a result, 2 specimens of each graft group could be implanted in each goat. After the implantation procedure, the animals received analgesics and antibiotics for 3 days (subcutaneous ampicillin (15 mg/kg/48 h; 96 h), buprenorfine (0.3 mg/12 h; 24 h), flunixine (75 mg/24 h; 72h)). Fluorochromes were administered at 4 weeks (tetracycline (1,000 mg/24 h; 72 h)), 8 weeks (calcein green (1,250 mg/24 h; 72 h)), and 12 weeks (alizarin (1,250 mg/24 h; 72 h)).

All procedures were approved by the Animal Ethics Committee of the University of Nijmegen (registration number 21034).

### Evaluation

The goats were killed 12 weeks after surgery, with an overdose of pentobarbital. Bone chambers were harvested with surrounding cortex and fixed in 4% buffered formalin. After 3 days, the contents were removed from the bone chambers and fixed for another 5–7 days. Undecalcified serial slices 40 μm in thickness were made parallel to the longitudinal axis of the chamber. 3 sections were used for histological evaluation: 1 central section and 2 peripheral sections 300 μm from the central section. Maximum bone ingrowth distance was defined as the largest distance between the bottom of the bone chamber and new bone in the graft cylinder, measured parallel to the longitudinal axis of the specimen. Maximum bone ingrowth distance of a specimen was calculated as the average of the maximum bone ingrowth distances in all 3 sections. Fluorescence microscopy was used to observe time dependence of bone ingrowth. After fluorescence microscopy, bone was coloured with a Goldner staining (green) or HE staining (pink) and acid phosphatase staining (osteoclasts). Bone ingrowth distances were measured with interactive computer-controlled image analysis. Backscatter electron imaging in combination with energy-dispersive spectroscopy (BEI-EDS) was used to identify and characterize microparticles.

### Statistics

The study was designed to detect a difference in bone ingrowth distance of 1 mm (equivalent to the observed standard deviation in previous studies) with an α of 0.05 and a power of 0.80 in a paired study design, corrected for double implantation in every goat. 2 chambers from the same experimental group were implanted in every animal. The mean of the 2 maximum ingrowth distances in these 2 chambers was calculated, to be used for statistical analysis. The data did not show a Gaussian distribution. Repeated-measures analysis of variance on ranks (Friedman test) was used in combination with the Student-Newman-Keuls post-hoc multiple comparisons method to identify significant differences between the graft groups (SigmaStat 3.5; Systat Software, Chicago, IL). Results for maximum bone ingrowth distance were expressed in mm as median and standard deviation (SD). The median values and tenth, twenty-fifth, seventy-fifth, and ninetieth percentiles were all used for box plot comparison.

## Results

### Impaction procedure

**Figure 2.**
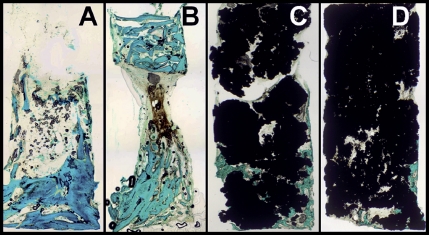
Shape of the bone ingrowth front in empty chambers (A), allograft with graft remnants at the top (B), non impacted, coated titanium particles (C), and coated, impacted titanium particles (D) (Goldner staining).

Impacted allograft specimens showed a homogeneous matrix of dense and efficiently packed bone grafts. Non-impacted Ti particle specimens showed inefficient packing at the periphery of the graft cylinder as a result of the relatively large diameter of the particles compared to the diameter of the inner lumen, with inter-particle pore sizes ranging from 10 to 500 μm. Impaction of Ti particles reduced intra-particle pore size by deformation of individual particles as well as reducing inter-particle pore size by more efficient packing of particles. This resulted in a homogeneous and dense matrix, especially at the central part of the graft cylinder where pore sizes ranged between 10 and 100 μm. The periphery of impacted Ti particle cylinders still contained areas with larger-sized pores, due to inefficient packing (Figure 2). Ingrowth holes of the bone chamber were often obstructed by individual Ti particles, both in non-impacted and in impacted Ti particle specimens.

### Surgical procedure and specimen retrieval

Implantations were technically uneventful. One goat died 5 days after operation, from an intestinal Clostridium infection complicated by sepsis. One goat suffered from a unilateral superficial wound infection, which was successfully treated by drainage and intramuscular antibiotics. Post-mortem radiographs showed unchanged implant positions and cortical thickening. During harvesting of the specimen from the lumen of the bone chamber, 4 specimens were lost from initially empty chambers and 2 specimens were lost from allograft-filled chambers due to the fragility of these specimens. Ti particle cylinders showed good integrity and were not damaged during harvesting. As a result, 18 “empty” specimens, 20 “bone” specimens, and 22 specimens from every Ti particle group were available for histological evaluation.

### Evaluation

Fluorochrome labeling showed only small areas of tetracycline-marked bone near the ingrowth openings or at the bottom of the chamber, which probably meant that the distant parts of bone ingrowth occurred after 4 weeks of implantation of the bone chambers. Ti particle groups and empty chambers showed broad apposition bands of calcein and narrow bands of alizarin, whereas apposition bands of calcein and alizarin in allograft specimens were more evenly distributed. In empty bone chambers and bone chambers filled with Ti particles, bone first invaded along the osteoconductive wall of the chamber. This peripheral bone ingrowth pattern was more pronounced in impacted Ti particles than in non-impacted Ti particles and empty chambers, and it was often not possible to determine a bone ingrowth front as in previous bone chamber studies. A clear bone ingrowth front was only observed in allografts. It was cone-shaped, in contrast to the other groups where the maximum ingrowth distance was located at the center of the graft cylinder (Figure 2).

**Figure 3.**
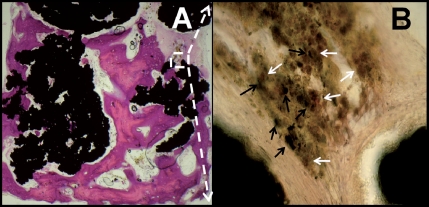
A. Osteoconduction of titanium particles with bone-surface contact (HE staining). B. Acid phosphatase staining (400×) with titanium microparticles (black arrows) engulfed by macrophages (solid white arrows).

HE staining of some coated and non-coated Ti particle specimens suggested areas of direct contact between invading bone and the surfaces of the Ti particles (Figure 3). In all 4 Ti particle groups, higher-magnification views (400×) of acid phosphatase-stained specimens showed dense microparticles engulfed by macrophages at contact areas between Ti particles. Corresponding to observations by light microscopy, BEI-EDS revealed titanium microparticles of irregular shape (diameter range 1–50 μm). There were no signs of increased osteoclast numbers or macrophage-associated osteolysis (Figure 3). No traces of silicium could be found by BEI-EDS, suggesting that the Bonitmatrix coating had been fully resorbed within 12 weeks of implantation. Beside invading bone, abundant ingrowth of fibrous tissue was seen throughout the whole graft cylinder (by HE staining). Fibrous armoring was also observed in impacted allograft cylinders—but to a lesser extent, which corresponds to inferior graft stability during harvesting compared to Ti particle cylinders.

**Figure 4.**
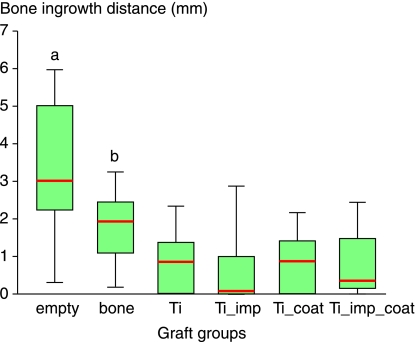
Box plot of graft groups and corresponding maximum bone ingrowth distances 12 weeks after implantation. Originally, empty bone chambers were more osteoconductive than bone chambers filled with osteoconductive bone or Ti particles (a, p ≤ 0.01). Bone particles were more osteoconductive than Ti particles (b, p ≤ 0.001).

Maximum bone ingrowth distances were higher in originally empty bone chambers (mean 3.2 (SD 1.9)) than in all other groups (p ≤ 0.01). Bone ingrowth distances were intermediate in allograft bone chips (mean 1.9 (SD 1.0)) but still twice as high as in non-impacted Ti particle groups (mean 0.9 (SD 0.8); p < 0.001) (Figure 4). Ti particles showed lower bone ingrowth distances after impaction. Application of the silicated calcium phosphate coating partially compensated for the negative effect of impaction (mean ingrowth distance of 0.1 (SD 1.1) for non-coated, impacted Ti particles as opposed to 0.4 (SD 0.9) for coated, impacted Ti particles). However, the effects of impaction and of application of a coating did not reach statistically significant levels.

## Discussion

Osteoconduction by the bone chamber is sensitive to inhibition by blockage of ingrowing bone by inserted graft materials. This hypothesis is supported by impairment of bone ingrowth by impaction (Tägil and Aspenberg 1998) and the observation that OP-1 increases bone ingrowth rates in impacted allograft cylinder by accelerated graft resorption (Jeppsson et al. 2003). The findings of our study are in accordance with these observations. However, some potential drawbacks of our study should be mentioned. First, the bone ingrowth front appeared to be highly variable with Ti particles and was often interrupted by the presence of the Ti particles in the center of the bone chamber lumen, especially in impacted Ti particle graft cylinders. Thus, maximum bone ingrowth distance was chosen instead of the more frequently measured mean ingrowth distance (Aspenberg et al. 1996). Secondly, non-impacted allografts were abandoned as a control group. Instead, empty chambers were chosen to exclude “allograft quality” as a potential confounder. Thirdly, 6 implants per tibia were used instead of 2 or 3. Fourthly, a Goldner stain was used instead of hematoxylin and eosin staining, which shows more cytological detail. An additional hematoxylin and eosin staining with acid phosphatase was used in several specimens of all graft groups to identify adverse reactions such as osteolysis.

In a previous study, larger and more porous Ti particles behaved mechanically superior to allograft bone particles (Walschot et al. 2010). In our study, all Ti particle graft cylinders showed abundant fibrous armoring, which further increases graft layer strength (Tägil and Aspenberg 2001). Correspondingly, during harvesting from the implanted bone chambers, the mechanical integrity of Ti particle cylinders was superior to that of impacted allograft cylinders. Large structural allografts show only peripheral invasion by host bone (Hooten et al. 1996) and require additional fixation by a cage for long-term stability (Gross and Goodman 2004). The key factor for long-term stability of bone impaction grafting reconstructions therefore seems to be the armoring by ingrowth of host bone throughout the whole allograft layer (van der Donk et al. 2002). In this respect, the small bone ingrowth distances in all Ti particle groups appear to be the main point of interest in this study.

All materials in the study were rinsed to remove chemical pollution. Bone ingrowth patterns in the allograft and originally empty control groups corresponded to previous observations (Aspenberg et al. 1996). Furthermore, although the bone chamber is a non-loaded model that might not be ideal for provocation of particle disease-mediated osteolysis, no signs of microparticle disease such as histiocytosis, lining of bone tissue by macrophages and/or osteoclasts, accumulation of giant cells, or toxic effects on fibroblasts (Mostardi et al. 2002, Warashina et al. 2003) were observed.

A mechanical rather than a biological factor seems to be responsible for the observed discrepancy between the already proven good osteoconductive properties after loose packing during intramedullary femoral application (Alffram et al. 2007, Turner et al. 2007) and the rather small bone ingrowth distances in more efficiently packed Ti particles in the bone chamber in this study. Osteons can grow into holes with a diameter of less than100 μm. However, the consensus seems to be that the optimal pore diameter for ingrowth of mineralized bone lies between 100 and 400 μm (Itälä et al. 2001). Intra-particle pores in small Ti particles have a diameter range of about 10–100 μm, and most intra-particle pores have a diameter of < 50 μm. Furthermore, small-sized Ti particles also have small inter-particle pores which are even further compromized by some degree of impaction during insertion of the Ti particles in the bone chamber, and during subsequent deliberate impaction. As opposed to allograft bone, the matrix of porous Ti particles is not resorbable. Mechanical obstruction by narrow intra-particle pores, but also by narrow inter-particle pores, could explain why the insertion of even non-impacted porous Ti particles—which are made of the same material as the bone chamber itself—impaired the good intrinsic osteoconductive properties of the titanium bone chamber to a larger extent than tightly packed allografts. The impairing effect of impaction of Ti particles on the ingrowth of bone through the already small inter-particle pores could only partially be compensated for by the rather thin (5–10 μm) and fully resorbed calcium phosphate coating, which itself was not expected to exert a significant obstructive effect. The observations from a pilot study in 3 goats with cemented acetabular revision reconstructions made of considerably larger, non-coated Ti particles (2.8–4.0 mm in diameter) are in agreement with our “inter-particle pore obstruction theory”: the corresponding macro-porous graft layers did indeed show local bony invasion throughout the whole graft layer with osseointegration (unpublished data).

In conclusion, Ti particles showed good fibrous armoring but inferior osteoconduction compared to allograft bone, especially after impaction. In this study, the small Ti particles tended to obliterate the lumen of the bone chamber. Implantation of larger-sized porous Ti particles in bone defects, with a clinically relevant degree of impaction, could offer an alternative to the bone conduction chamber model to evaluate bone ingrowth in a reconstructive layer of porous Ti particles. A realistic in vivo study should be conducted in order to evaluate the potentially harmful effects of titanium microparticles generated during impaction of Ti particles.
